# Failure in the mother-young communication in domestic mammals: endocrine and behavioral aspects

**DOI:** 10.3389/fvets.2025.1589916

**Published:** 2025-05-29

**Authors:** Daniel Mota-Rojas, Cécile Bienboire-Frosini, Arthur Fernandes Bettencourt, Dina Villanueva-García, Adriana Domínguez-Oliva, Adolfo Álvarez-Macías, Vivian Fischer, Patricia Mora-Medina, Adriana Olmos-Hernández, Ismael Hernández-Avalos, Julio Martínez-Burnes, Ayman H. Abd El-Aziz, Agustín Orihuela, Temple Grandin

**Affiliations:** ^1^Neurophysiology, Behavior and Animal Welfare Assessment, DPAA, Universidad Autónoma Metropolitana (UAM), Mexico City, Mexico; ^2^EPLFPA-Avignon, Site Agroparc, Avignon, France; ^3^Department of Animal Science, Federal University of Santa Maria, Santa Maria, Brazil; ^4^Postgraduate Program in Animal Science, Federal University of Rio Grande do Sul, Porto Alegre, Brazil; ^5^Division of Neonatology, Hospital Infantil de México Federico Gómez, Mexico City, Mexico; ^6^Department of Animal Science, Federal University of Rio Grande do Sul, Porto Alegre, Brazil; ^7^Facultad de Estudios Superiores Cuautitlán, FESC, Universidad Nacional Autónoma de México (UNAM), Cuautitlán, Mexico; ^8^Division of Biotechnology-Bioterio and Experimental Surgery, Instituto Nacional de Rehabilitación Luis Guillermo Ibarra Ibarra (INR-LGII), Mexico City, Mexico; ^9^Facultad de Medicina Veterinaria y Zootecnia, Instituto de Ecología Aplicada, Universidad Autónoma de Tamaulipas, Victoria City, Mexico; ^10^Animal Husbandry and Animal Wealth Development Department, Faculty of Veterinary Medicine, Damanhour University, Damanhour, Egypt; ^11^Neurophysiology and Animal Behavior, Facultad de Ciencias Agropecuarias, Universidad Autónoma del Estado de Morelos, Cuernavaca, Morelos, Mexico; ^12^Department of Animal Science, Colorado State University, Fort Collins, CO, United States

**Keywords:** maternal behavior, newborn rejection, dystocia, oxytocin, maternal care, maternal recognition

## Abstract

Mothering and bonding represent fundamental aspects of survival and development in domestic mammalian species. The mother-young interaction immediately after parturition is a critical event where the mother establishes selective care for the offspring, and the newborn responds to maternal stimulation. To develop this bond, maternal responses such as nest building, grooming, allowing suckling, or retrieval of the young need to be performed within the so-called sensitive period. This review discusses the factors that lead to failure in mother-young bonding in domestic mammals, analyzing mother- and young-related factors that might impair maternal recognition. Among these factors, endocrine aspects such as oxytocin impairments and lower release interfere with the expression of maternal behavior. Moreover, a complex network of hormonal regulators, including steroid hormones (estrogen, progesterone), prolactin, and dopamine, is required to modulate the parenting and attachment process. In addition, other biological aspects such as prenatal conditions, maternal nutritional state, parity, and environmental factors can affect the quality of maternal care. Regarding young-related factors, low vitality due to events such as meconium aspiration syndrome decreases the newborn’s motivation to interact and develop the mother-young bond. Recognizing these aspects to prevent offspring rejection is essential to neonatal survival. Peripartum monitoring and precision livestock farming are suggested methods to ensure appropriate mother-young communication.

## 1 Introduction

**Figure 1:**
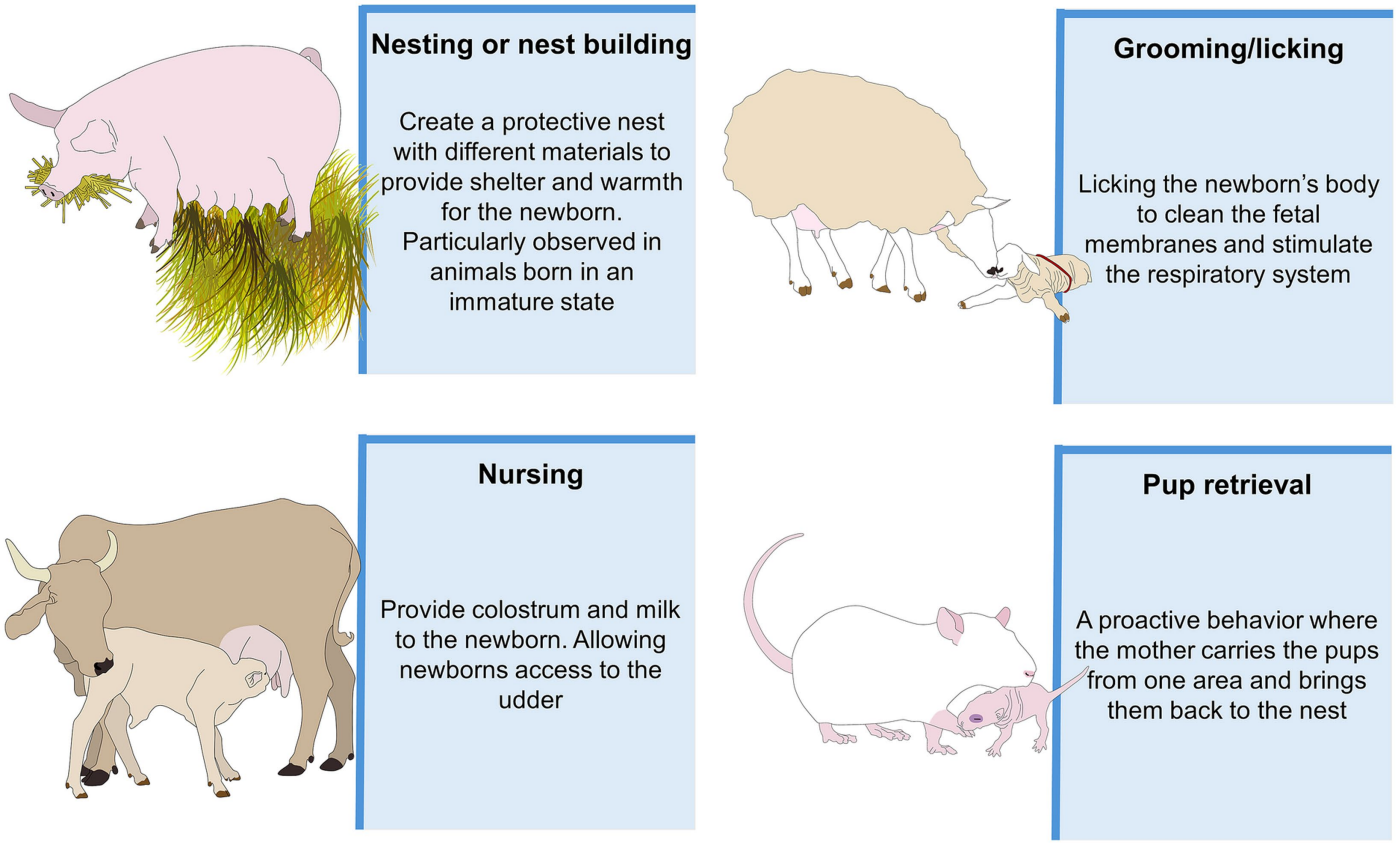
Representative examples of key maternal behaviors discussed in this review. The images illustrate a range of caregiving actions typically observed during the early postnatal period, including nursing posture, grooming, and close physical contact.

In domestic animals, including birds and mammals, maternal care refers to behaviors that ensure and support the offspring’s well-being, survival, development, and growth, as shown in Figure 1, where the main maternal behaviors in mammals are summarized (1–4). Although domestic mammals can respond maternally from puberty through adulthood, the intensity of maternal responses increases at parturition (5). Immediately after parturition, the mother displays several behaviors to nurse, protect, and stimulate the newborn through olfactory, tactile, auditory, and vocal cues (6–8). These stimuli elicit neuroendocrine and behavioral responses in females (7–10). Changes in plasma estradiol, progesterone, and prolactin concentrations start during gestation to prepare the maternal behaviors before parturition (11). Estradiol, in particular, is critical for activating parenting-related neural circuits in key brain regions, such as the medial preoptic area (MPOA). Also, estrogen and progesterone work in tandem to prepare female mammals for parenthood (5). The importance of the different types of stimuli in maternal behavior depends on the species. For example, smell is critical for ruminants to start the interaction between the newborn and the mother (12, 13), while in rodents, tactile and auditory cues (e.g., ultrasonic vocalizations) are needed for maternal recognition (14, 15). The quality of maternal care significantly influences the offspring’s survival (4, 16). Therefore, factors jeopardizing communication between the newborn and the mother directly affect their welfare.

The interaction between the mother and the newborn is a selective learning process to establish the mother-young bond, an event observed in all mammals (6, 17–20). This process is promoted by maternal behaviors such as nest building, licking or grooming, allowing suckling or crouching to nurse, retrieval of the young, milk provision, protection from predators, and warmth (1, 2, 5, 21). The first hours after parturition are essential to consolidate maternal responsiveness towards the newborn (22). This is the sensitive period, where selectivity to filial offspring is established and sustained (23–25). In domestic mammals, maternal selectivity is a learning process where the mother rapidly establishes an exclusive bond with her offspring to provide exclusive care (26, 27). After establishing this bond, the mother generally rejects nursing any non-filial offspring (28). This process occurs following contact with the newborn due to sensory stimulation through olfactory, tactile, auditory, and visual cues (29, 30). Olfactory and tactile stimulation comes from the females’ attraction to the amniotic fluid and fetal membranes in the newborn’s body, encouraging sniffing and licking (7, 31, 32). The presence and proximity of the newborn contribute to the visual and auditory (e.g., vocalization) cues needed to recognize her young (26). Cross-species adoption has been documented in several taxa, including canids and rodents (33, 34). For further insights into foster parenting in both wild and domestic species, the article by Jain and Shakarad (35) is recommended. During the bonding process, hormonal and physical aspects promote the cross-modal sensory recognition between the mother and the newborn (7, 14, 36). Neurochemically, oxytocin (OXT) is the main neuropeptide modulating parturition and lactation, as well as maternal and social behavior (37–39). Studies in OXT knockout animal models have reported that the lack of OXT or expression of OXT receptors (OXTr) in the mother affects the presentation of maternal behaviors (40, 41).

Maternal care and the establishment of the mother-offspring bond can be affected by several factors related to the mother or the young. Examples of maternal-related aspects are hormonal imbalances, maternal experience, temperament of the mother, and birth type (e.g., dystocia) (42, 43). Behavioral disruptions in mother-young interactions can also stem from environmental stressors. For instance, corticotropin-related factor (CRF) is a neuropeptide involved in coordinating stress responses and elevated CRF concentrations have been associated with impaired maternal behaviors. In rodent models, increased CRF activity in the bed nucleus of the stria terminalis correlates with maternal neglect, suggesting that heightened stress responses can override nurturing behaviors (44). Sawalha et al. (45) mention that poor maternal care in sheep is directly related to newborn death. Moreover, young-related issues can be associated with hypothermia, hypoxia, and meconium aspiration syndrome, which might result in low neonatal vitality (46). These events might result in abnormal responses, including failure to groom the newborn, aggression towards the offspring, delay in colostrum provisioning, and even abandonment (1, 47, 48).

For veterinarians, researchers, and farm managers, encouraging an appropriate mother-young bonding is related to the animal’s fitness and productive performance —neonatal mortality and morbidity risk decrease when the offspring receives proper maternal care (4). Moreover, for females, it has effects on their health, and some authors even refer to consequences related to emotional distress (49). Thus, as the failure to establish a mother-young bond has consequences on both ends, the present review aims to discuss the factors that lead to a failure in the mother-young bonding, analyzing mother- and young-related factors that might impair maternal recognition in domestic mammals.

## 2 Search methodology

**Figure 2:**
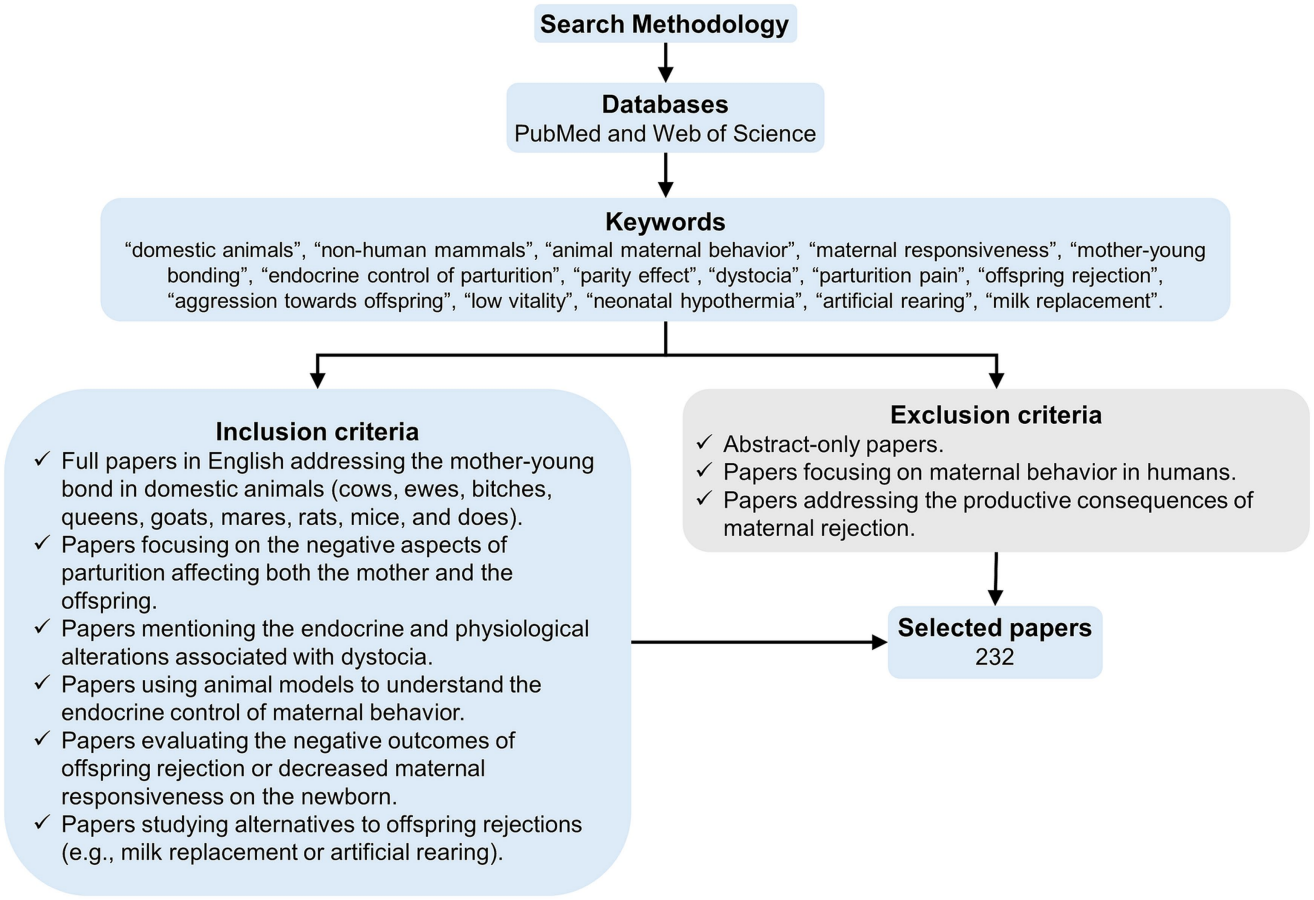
Search methodology.

PubMed and Web of Science were the databases used for searching, as both platforms cover numerous multidisciplinary indexed journals. Combinations of the keywords shown in Figure 2 were used to search for papers. As the present review focuses on domestic mammals, only those papers about domestic species were included. Duplicates were removed when selecting the papers. Selected articles covered the endocrine, physiological, behavioral, and environmental factors influencing the mother-young bond. Those that addressed newborn rejection and the adverse effects of it were also considered. Papers addressing wild species or humans were excluded.

## 3 Mother-related aspects of impaired maternal bonding

### 3.1 Oxytocin as a mediator of maternal behavior

Oxytocin (OXT) is the main modulator of maternal responses during the peripartum period (38, 40, 50). It contributes to parturition, lactation, and social interaction, and reduces stress/anxiety during these periods (6, 51, 52). In domestic mammals, OXT is released by the hypothalamic paraventricular nucleus (PVN) (53, 54), and expression of OXT receptors (OXTr) after parturition is mainly observed in the PVN, supraoptic nucleus (SON), bed nucleus of the stria terminalis (BNST), and lateral septum (LS) (40, 41, 55). OXT acts on cerebral regions such as the medial preoptic area (MPOA), ventral tegmental area (VTA), and nucleus accumbens (6, 53). In ewes (*Ovis aries*), Da Costa et al. (56) found high concentrations of OXT in the PVN at lambing (up to 250 pM).

The release of OXT and OXTr expression is closely related to the exchange of physical and tactile stimuli between the mother and the newborn, and the offspring’s presence reinforces and strengthens the mother-young bond (14). This was reported by Francis et al. (57) in Long-Evans rats (*Rattus norvegicus*). In these animals, OXTr levels were higher in females who actively licked/groomed and adopted an arched-back nursing posture. Thus, adequate concentrations of OXT at parturition are required to facilitate maternal behavior, as observed in rats, in whom administration of OXT antagonists into the MPOA and VTA delayed the onset of maternal care (6).

The importance of OXT and OXTr in rodents has been studied with knockout models where the females lack the expression of receptors or OXT release. Rich et al. (40) studied the effect of impaired OXT signaling in total body and forebrain knockout mouse (*Mus musculus*) models on maternal behavior. The authors found that dams with total body knockout of OXTr had significantly higher levels of pup abandonment (66.6% of dams abandoned the litter and performed cannibalism) at 1-day post-parturition. Similarly, higher mortality rates have been observed in OXTr knockout mice, with 40% of dams having total pup mortality (41). Other studies reported that OXTr knockout mice decreased pup retrieval and high plasma corticosterone concentrations (up to 300 ng/mg), while OXT elicits alloparental care of newborn pups (58). Similarly, Pedersen et al. (59) observed decreased pup recovery and survival in OXTr knock-out mice.

**Figure 3:**
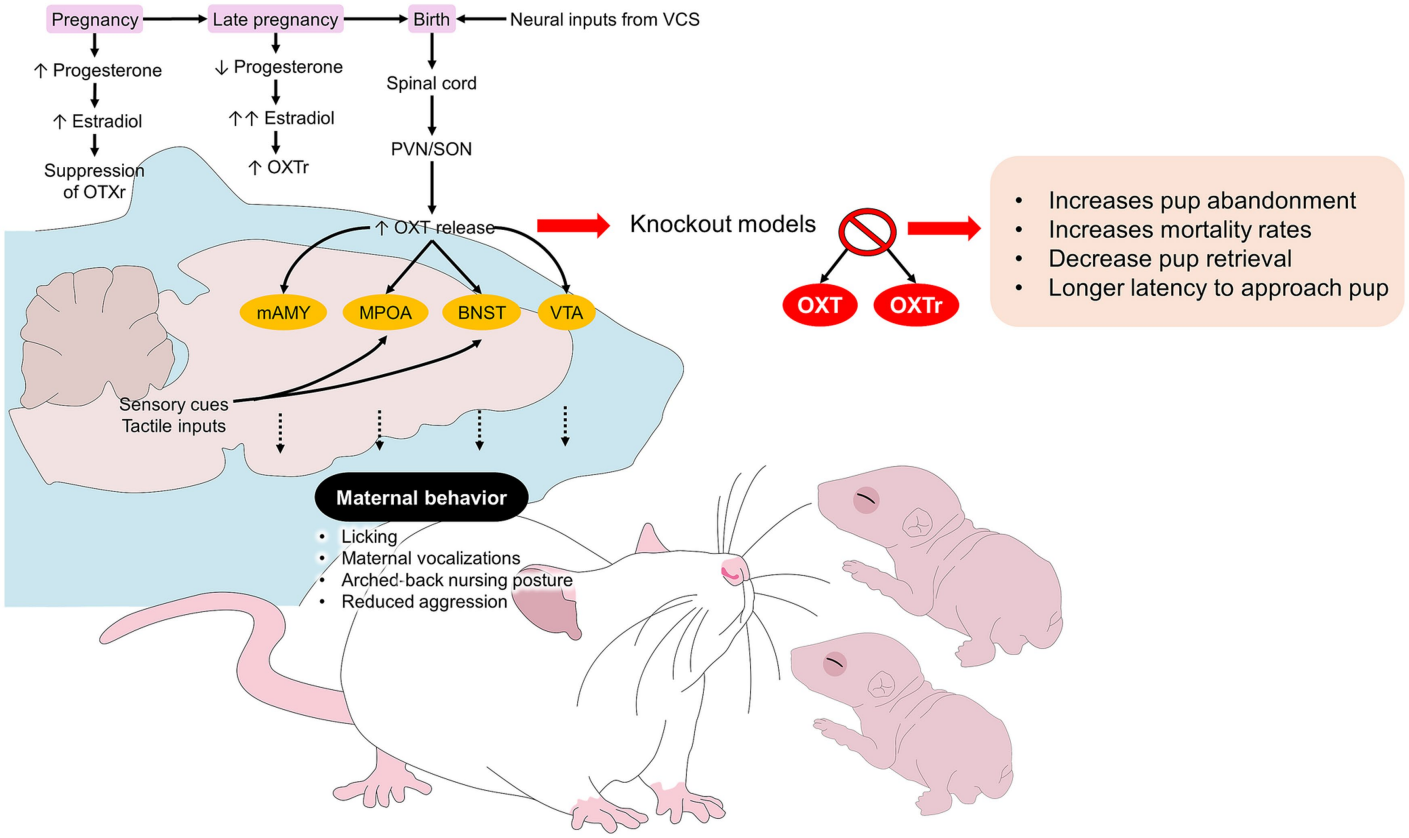
Endocrine modulation of maternal behavior and behavioral alterations in murine knockout models. Maternal care, including licking, calls, arched-back posture to nurse, and reduced aggression, are behaviors elicited by the release of OXT in the PVN and SON and its action on the mAMY, MPOA, BNST, and VTA. However, in knockout models, where there is no release of OXT or a lack of OXTr expression, abnormal behaviors such as pup abandonment, increased mortality rates, decreased pup retrieval, and aggression are developed. BNST, bed nucleus of the stria terminalis; mAMY, medial amygdala; MPOA, medial preoptic area; OXT, oxytocin; OXTr, oxytocin receptor; PVN, paraventricular nucleus; SON, supraoptic nucleus; VCS, vaginal cervical stimulation; VTA, ventral tegmental area.

Maternal response to the newborn presence is also mediated by OXT as Marlin et al. (60) reported in female mice. in female mice. It was found that, during pup calls, OXT and OXTr enhanced pup retrieval via the activation of the left auditory cortex. This was also mentioned by Carcea et al. (53), who concluded that OXT is required to facilitate neuroplasticity in the auditory cortex so the female can recognize distressed pup calls (60). Similarly, experimental models in rodents have found that OXT incites maternal calls to retrieve pups. In this sense, intranasal administration of OXT (0.8 IU/kg) increased the frequency of maternal ultrasonic vocalizations (above 50 kHz) when reuniting with their pups (1.5 sweeps/s), in contrast to control females (0.5 sweeps/s) without OXT administration. Moreover, female mice without OXT administration had longer latency times to approach the pups and lower rates of pup retrieval than females receiving OXT, aspects that compromise maternal care (Figure 3) (5, 16, 61).

**Figure 4:**
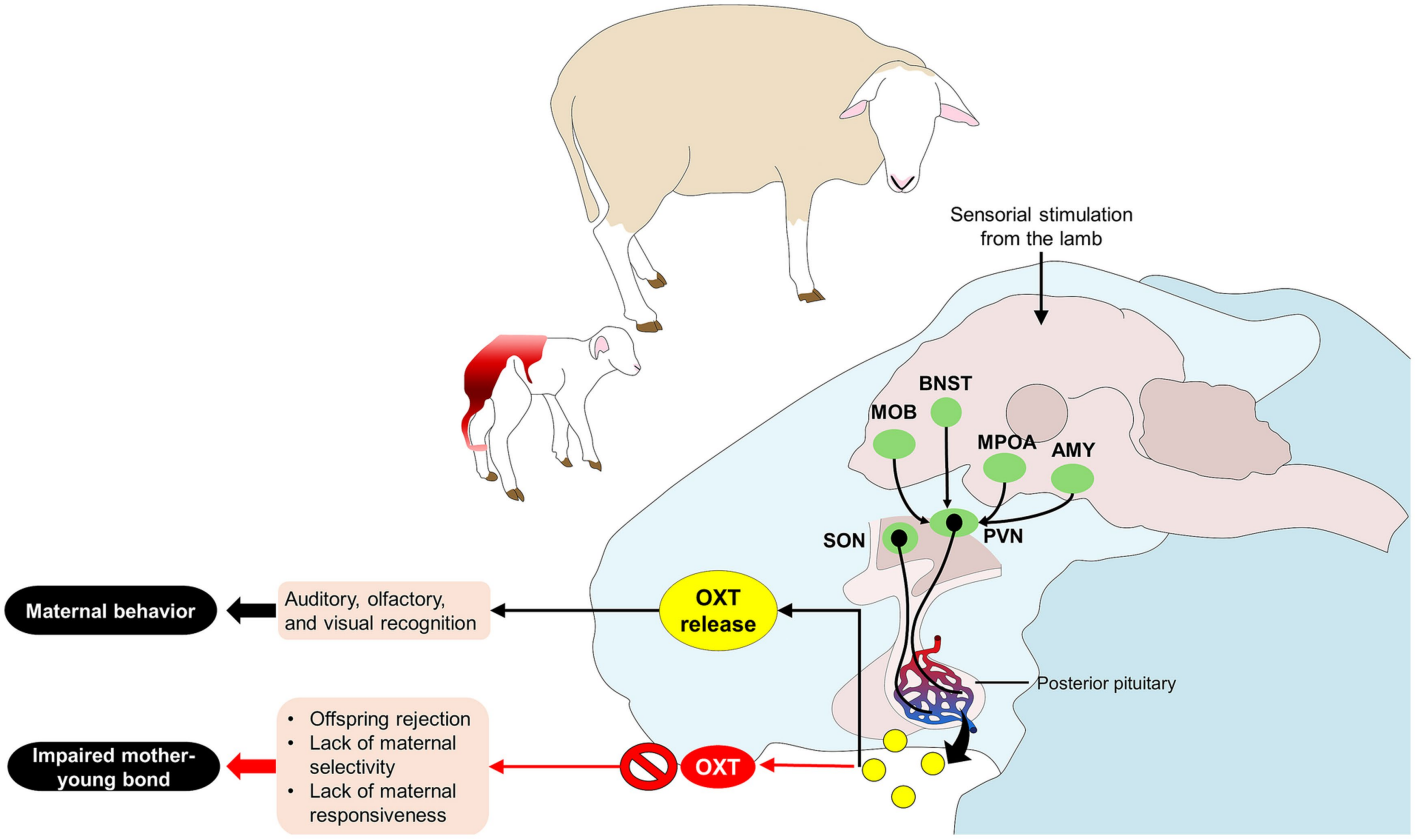
Modulation of maternal behavior and impaired mother-young bonds. Sensorial stimulation from the lamb activates key structures such as the MPOA. The MPOA has projections to the pituitary gland, where OXT is released due to the activation of the PVN and SON. Additionally, connections to other cerebral regions (e.g., MOB, BNST, and AMY) promote the newborn’s auditory, olfactory, and visual recognition, motivating maternal behavior. Contrarily, when OXT release is inhibited, the mother-young bond does not develop, and behaviors such as offspring rejection and lack of maternal responsiveness are observed. AMY, amygdala; BNST, bed nucleus of the stria terminalis; MOB, main olfactory bulb; MPOA, medial preoptic area; OXT, oxytocin; PVN, paraventricular nucleus; SON, supraoptic nucleus.

In ewes, Kendrick et al. (62) reported intracerebroventricular administration of OXT to ovariohysterectomized animals and its effect on maternal behaviors such as low-pitch bleats, sniffing, licking, and following the lamb. The results showed that control animals without OXT administration had a lower frequency of said behaviors than animals receiving 5–20 μg of OXT. Similarly, infusions of OXT in the PVN induced maternal behavior in 75% of ewes (56). Lévy et al. (63) mention that OXT release within the main olfactory bulb is necessary to establish the olfactory recognition of the offspring in ewes. Also, disruptions in OXT signaling can impair maternal behaviors; for example, in sheep, blocking OXTr hinders the development of maternal bonding behaviors (30). Mother-young bonding is essential for the young’s survival and future social development. These studies show that OXT is needed to develop maternal bonding and that the interaction of cerebral areas and the activation of OXTr are necessary to facilitate maternal responsiveness (Figure 4) (26, 27, 64, 65).

In dairy cattle (*Bos taurus, Bos indicus*), Neave et al. (66) observed that salivary OXT concentration tended to be higher (232.4 vs. 182.5 pg./mL) in cows that had full contact (23 h) with their calves compared to cows that had partial contact (10 h). Regarding the weaning practice adopted, the authors also observed a trend for higher salivary OXT concentration in cows with unchanged contact (between weeks 8 and 10) compared to cows that experienced reduced contact with the calf during the same period (235.8 vs. 179.8 pg./mL). These findings were attributed to the cow-calf bond through a trend of positive association between the proportion of total daily time spent together touching (in contact, including sniffing and cleaning) and salivary OXT concentration around nursing. However, Geburt et al. (67) do not suggest the adoption of salivary OXT as a valid biomarker for maternal behavior in cattle. Further studies with salivary OXT are needed since, unlike plasma, the OXT peak in saliva is not yet known (66).

### 3.2 Other biological mediators

Beyond oxytocin, other hormones are involved in the mother-young bonding. As related by Champagne and Curley (68), the influence of hormones on maternal behavior has been rigorously examined through various experimental protocols in rodents. A common method involves assessing the latency period for virgin female rats to exhibit maternal behaviors, such as retrieving and crouching over pups. The latency in days serves as a primary dependent measure for evaluating the effects of genetic, neuroanatomical, and pharmacological factors on maternal responsiveness. Estradiol (E2) treatment, particularly when combined with progesterone (P), significantly reduces the latency for the onset of maternal behavior. Additionally, administering prolactin (PRL) or OXT alongside E2 and P further enhances the promptness of maternal responsiveness. Experimental results indicate that ovariectomized and hysterectomized rats without hormonal supplementation exhibit the longest latency (5–7 days), whereas those treated with E2 and P, or with added prolactin or oxytocin, show reduced latencies of around 2 days. These findings in laboratory settings demonstrated the crucial role of specific hormonal combinations in facilitating maternal behaviors.

Indeed, E2 was shown to increase the expression of OXTr in the brain, thereby enhancing the effect of OXT on maternal behavior (69). E2 promotes maternal behavior through changes in the estrogen receptor alpha (ERα or ESR1). For example, (i) silencing ESR1 expression in the MPOA significantly reduced pup care in mice; (ii) an increased expression of ERα cell density is observed in subsequent pregnancies in ewes (5).

Also, in mammals, PRL plays a crucial role in maternal behavior, particularly in facilitating the onset of maternal behaviors and the care of young. Studies showed that PRL acts on the MPOA to promote maternal behaviors and lactation (5, 70). Other mediators, such as dopamine, inhibit fear pathways related to the offspring and activate reward-related responses to the newborn, which increases the presentation of maternal behaviors (68). Table 1 summarizes the main biological mediators related to maternal behavior in different species.

**Table 1:** Mediators of maternal behavior according to the species.

Mediator	Species	Role in mother-offspring behaviors	Reference
Cortisol	Ewes	Increases in salivary cortisol are related to increased grooming behavior, but impaired ability to follow their lamb	(211)
	Goats	Goats with higher salivary cortisol concentrations displayed less grooming and nosing	(212)
	Sows	Lower hair cortisol concentrations are related to increased maternal behaviors	(213)
Corticosterone	Mice	High doses of corticosterone (40 mg/kg) reduce time spent in the nest and nursing	(214)
	Rats	Adrenalectomized rats had lower expression of licking and time in the nest. In contrast, adrenalectomized rats receiving low corticosterone doses (25 μg/mL) maintained maternal behaviors	(215)
Dopamine	Ewes	Dopamine concentrations increase during lambing. Ewes with twin lambs have higher dopamine concentrations than animals with single lambs and maternal behaviors of better quality (suckling, grooming, and following)	(45)
	Mares	In non-parturient mares, administration of a combination of estradiol + progesterone + dopamine increased preference for their foal regardless of vaginocervical stimulation	(216)
	Mice	Dopamine is required to motivate pup retrieval at the onset of pup contact	(217)
	Rats	Dopaminergic projections interact with OXT for offspring recognition due to olfactory cues. Moreover, dopamine inhibits fear circuits and motivates the female to approach the newborn and perform licking, grooming, and pup retrieval	(218)
Endocannabinoid system	Mice	Blocking receptors of the endocannabinoid system (CB1) impairs mother-pup interactions. Treated females perform less crouching over their pups and delay pup retrieval	(219)
	Rats	Administration of cannabinoid antagonists decreases maternal behavior, as observed by less time spent licking the pups and increased feeding time in the female	(220)
Estradiol	Cattle	Increases in plasma estradiol (together with increases in prolactin, cortisol, and activation of the oxytocinergic system) modulate the interest of the dam to the newborn calf	(1)
	Bitches	High concentrations of estradiol participate in the onset of maternal behaviors (pup recognition, nursing)	(221)
	Ewes	Increases in plasma estradiol and vaginocervical stimulation, increase maternal responsiveness and expression of OXTr	(54)
	Goats	Estrogens are associated with the onset of licking and accepting the newborn	(26)
	Rats	Administration of steroids to nulliparous rats stimulates a fast onset of maternal behaviors	(222)
Noradrenaline	Ewes	Higher concentrations are related to better maternal behavior (suckling, grooming, and following)	(45)
	Mice	Disruption of norepinephrine and epinephrine-producing genes inhibit pup retrieval and increase pup mortality	(223)
Opiods	Ewes	Morphine administration reduces lamb rejection, while morphine+corticotrophin-relesasing factor potentiates lamb acceptance	(224)
	Rats	Morphine administration disrupts maternal behavior in primiparous rats. Impairs pup retrieval and crouching over the pup	(225)
Progesterone	Rats	Stimulate sniffing, pup licking, retrieving, huddling pups together, crouching over the pups, and nursing. It also inhibits the neophobic response to pups	(226)
Prolactin	Dogs	High prolactin concentrations during the peripartum period contribute to the onset of licking/grooming the pup, nursing, and increasing the time spent in contact with the newborn	(227)
	Ewes	High prolactin concentrations are not related to the onset of maternal behavior	(228)
	Mice	Reduction of prolactin in the pituitary gland inhibited behaviors such as pup retrieval and nursing	(229)
	Rats	Prolactin is related to the immediate onset of maternal behaviors (nest building, pup retrieval, and licking/grooming the pups)	(230)
	Sows	High prolactin concentrations in the pre-farrowing period motivate nest-building	(231)
Serotonin	Mice	Mutations that cause serotonin depletion cause maladaptive mothering. Lack of serotonin increases cannibalization and reduces pup retrieval, nest construction, and nursing	(232)
	Rats	Agonism to serotonin 5-HT_2A_ and 5-HT_2C_ receptors impair maternal behaviors such as pup preference and retrieval, nest building, and pup crouching	(233)

This multifactorial hormonal framework reflects the need for mammals to adapt to dynamic environments, where social and parental behaviors are crucial for survival and reproductive success.

### 3.3 Maternal experience: primiparous and multiparous females

Several studies on domestic mammals highlight maternal experience’s importance in ensuring an adequate mother-young bond (38, 51, 71, 72). Although maternal behavior is innate in mammals, the ability to care for the offspring increases with parity (51, 73). Primiparous mammals are more likely to be reactive or aggressive towards the newborn and even abandon them due to increased stress, anxiety, and neophobia (8, 74). Infanticide cases in non-lactating primiparous female rats are likely attributed to the lack of continuous feedback from the pups (75, 76). Indeed, the postpartum period requires decreased anxiety to accept the offspring and facilitate social bonding (40). Thus, high OXT concentrations during the postpartum period are required due to its anxiolytic effect, as Windle et al. (77) mentioned in rats, in whom intracerebroventricular infusion of OXT reduced anxiety and stress.

Females undergoing their first parity are exposed to parturition stress. Studies in small-tailed Han ewes have shown lower OXT concentrations (13 pg./mL) in primiparous ewes than multiparous females (17 pg./mL, 3 pg./mL less than primiparous) during the first-week post lambing (45), which, as discussed in the previous section, might be related to a better quality of maternal performance immediately after birth. When comparing YorkshireXLandrace gilts to sows (*Sus domesticus*) at sixth parity, it was found that gilts had a greater contraction (12.30 mmHg) and stillbirths due to the dystocic farrowing, causing neonatal mortality (78). Thus, inexperienced mothers do not show high maternal competence, resulting in negative maternal responses, lack of affiliative behaviors, giving birth to smaller offspring, and low milk production (54, 79).

Moreover, it is known that neonatal mortality is higher in primiparous mammals due to their maternal inexperience (80–83). Anwar et al. (73) reported the main risk factors for the mortality of lambs and kids (*Capra aeagrus hircus*). The main factor was poor maternal care, causing a mortality rate of 1.2/per 100 animals and an increased hazard for death (24.56 times). Insufficient nurturing behaviors (e.g., not cleaning the newborns, lack of nursing, or not providing warmth and protection) were linked to lower survival rates (36). Likewise, in Chinese Hu sheep, primiparous ewes had a higher incidence of lamb abandonment and lower lamb survival when compared to multiparous animals (47). This suggests that inexperienced mothers may take longer to initiate interactions with their offspring, which might influence the survival and welfare of newborn mammals (1).

The delay in starting maternal behaviors such as grooming was reported in primiparous Scottish blackface and Suffolk ewes by Dwyer and Lawrence (84), where primiparous animals had a latency to groom of 106 s, while parity 2, 3, and 4 recorded 39.8, 32.2, and 10.2 s. Moreover, first-parity animals expressed more maternal rejection, and lamb avoidance. In Corriedale ewes, while 100% of multiparous animals groomed the newborn, only 79.4% of primiparous females performed said behavior (85). In small-tailed Han ewes, Wang et al. (45) found that primiparous females had a higher incidence of udder refusal immediately after lambing (frequency of up to 1.3) than multiparous animals frequency of 1.0. Karaca et al. (42) compared the maternal behavior of primiparous and multiparous Norduz ewes, in the same species. Behaviors such as grooming were not affected by parity; however, primiparous animals showed aggression together with the prevention of sucking, butting, and low/high pitch bleating. Mean sucking duration was also shorter in primiparous ewes. Parity also affects the offspring behavior, as reported in small-tailed Han ewes and lambs, where lambs from primiparous animals suckled and attempted to stand later than multiparous newborns (86), suggesting that maternal experience can affect the mother-young relation.

Lévy et al. (87) also highlighted that neurochemical signaling in primiparous ewes is attenuated, possibly explaining why maternal behavior in nulliparous animals is lower than multiparous. When comparing the OXT concentrations in the olfactory bulb of Clun Forest ewes, it was found that primiparous had lower concentrations (up to 209 ± 45 pM) than multiparous animals (up to 401 ± 148.8 pM) (87). Likewise, the lack of olfactory stimulation (e.g., washing the amniotic fluids from the lamb’s coat) significantly reduced licking behaviors and acceptance at the udder and increased aggression towards the newborn lamb (88). Additionally, anosmia delayed the onset of licking and maternal bleats in primiparous ewes, while multiparous females did not present maternal disturbances (89). These findings suggest that, for primiparous females, sensory cues are critical to initiate maternal behaviors and that maternal experience can compensate for the lack of these signals. In ewes, another aspect that significantly affects the presentation of maternal behavior in primiparous animals is the administration of peridural anesthesia. This was reported by Krehbiel et al. (90), who found that 7/8 primiparous ewes did not have interest in their lamb within the first 30 min.

In goats, similar results were reported in West African Dwarf females, in whom latency to groom was longer in primiparous than multiparous animals, values that were related to lower vigor levels (91). Higher intensity of licking was also recorded during the first hour post-calving in multiparous Friesian dairy cattle, whereas heifers exhibited higher frequencies at two hours post-calving (92). In dairy goats, Cano-Suarez et al. (72) compared the behavior of primiparous and multiparous animals immediately after parturition. The findings showed that only 9% of primiparous kids were nursed within the first hour after partum, in contrast to the 33% of multiparous kids. Moreover, the latency to lick the newborns was also longer in primiparous goats (365.6 ± 188.56 s vs. 60.5 ± 24.09 s).

Maternal experience can also be obtained by exposing virgin mammals to newborns. Okabe et al. (51) found that repeated exposure to newborns decreased retrieving latencies in virgin female mice compared to females not frequently exposed to pups. Moreover, repeated pup exposure increased the proportion of OXT neurons and OXTr expressing c-Fos in the preoptic area (POA; up to 8%). The concentration of OXT also significantly increased in the POA (51). Therefore, as previously mentioned, OXT is also related to maternal experience, as females that are previously exposed to newborns improve their performance. Moreover, Carcea et al. (53) evaluated the effect of learned maternal care in virgin mice when housed with experienced mothers, which showed that virgin females learned pup retrieval and activated OXT neurons in the PVN.

Although most studies focus on the differences in the formation of the mother-calf bond between primiparous and multiparous mothers shortly after birth, there is evidence that the strength of the bond established can influence the physiological response of the animal months later in critical periods such as weaning in beef cattle. De Paula et al. (93) observed that physiological parameters such as total protein, albumin, hematocrit percentage, and albumin concentration indicated that the magnitude of stress after weaning at 7.5 months was greater in multiparous Nellore cows compared to primiparous and secundiparous ones. Thus, these studies show that, in general, primiparous females have a reduced maternal aptitude that might impair or delay the mother-young bonding. Dystocia and pain during parturition.

The birth process per se is a stressful event accompanied by pain and considerable discomfort (94). Physiological events such as uterine contractions, cervical dilation, and birth canal stretching are painful stimuli that are needed to start parturition (95, 96). However, when parturition is prolonged beyond the normal period for each species or non-progressive, dystocia and periparturient stress have long-term adverse effects on both the mother and the newborn (97, 98). The lack of oxytocinergic pathways during parturition might also influence the mother-young bond and peripartum stress as the presence of OXT is required to inhibit the expression of the corticotropin-releasing factor (CRF) at the PVN by GABAergic neurons (99).

In general, the causes of dystocia include the offspring’s size, fetal position, pelvic dimension, insufficient cervix dilation, uterine torsion, and vaginal prolapse (100, 101). Several studies have reported behavioral alterations and impaired mother-offspring interaction in dystocic domestic mammals. For example, Redfearn et al. (98) mention that dystocic ewes expressed lower maternal behaviors such as licking, bonding, and circling. These behaviors might be related to pain, a state that has a severe welfare impact on the mother’s health (102).

Regueiro et al. (85) found an extended expulsion phase (50.5 ± 4.2 min) in primiparous Corriedale ewes correlated with low maternal behavior scores (3.6 ± 0.2). In contrast, the expulsion phase of multiparous ewes lasted 32.2 ± 2.6 min and had maternal scores of 4.7 ± 0.1. Additionally, low Apgar (mnemonic for appearance, pulse, grimace, activity, and respiration) scores (7.7 ± 0.7) were recorded in lambs with longer lambing times, in whom also suckling behavior within the first two hours was affected, and only 53.9% of lambs performed. These results show that extended parturition time in mammals negatively influences the presentation of maternal behaviors.

**Figure 5:**
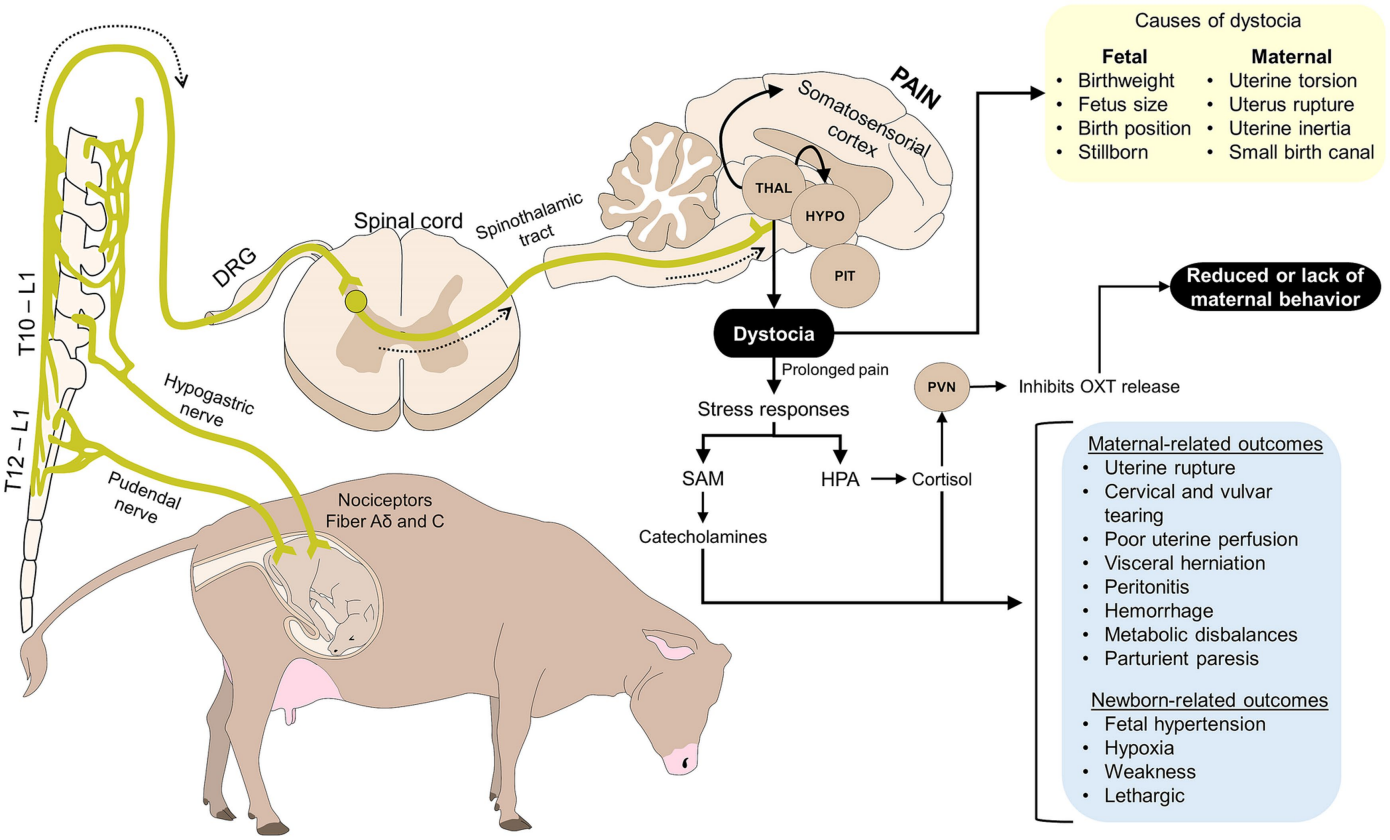
Effects of dystocia and pain on the mother-young bond. Prolonged pain, a characteristic of dystocia, causes several adverse effects on the mother and the offspring, which affect maternal care. Pain activates the HPA axis, leading to cortisol release. In the presence of cortisol, OXT release is inhibited, reducing maternal behavior. Moreover, dystocia has other maternal consequences, such as uterine rupture, poor uterine perfusion, hemorrhage, and parturient paresis, among others. These alterations cause exhaustion or movement impairment in the mother, disrupting the mother-young bond. DRG, dorsal root ganglion; HPA, hypothalamic–pituitary–adrenal axis; HYPO, hypothalamus; L, lumbar vertebrae; OXT, oxytocin; PIT, pituitary; PVN, paraventricular nucleus; SAM, sympathetic-adrenomedullary axis; T, thoracic vertebrae; THAL, thalamus.

Dystocia is also associated with an extended perception of pain during parturition, a process that is exacerbated by vaginocervical stimulation and uterine contractions (27, 103, 104). Figure 5 schematizes the effects that pain causes on maternal responsiveness (8, 100, 105). One of the main mechanisms involved in mother-young bond disruption is the hypothalamic–pituitary–adrenal axis activation and cortisol release, inhibiting OXT secretion (105). Additionally, parturition stress releases catecholamines, which cause fetal hypertension and poor uterine perfusion (106, 107).

As previously mentioned, when OXT is not available at birth, or its concentrations decrease, maternal behavior is altered, and females often reject or avoid their offspring due to extreme exhaustion from the prolonged delivery (108, 109). For example, in dogs (*Canis lupus familiaris*), Uchańska et al. (110) reported that pain reduces maternal instinct. In sheep and goats, Ring (111) mentions that prolonged delivery results in exhaustion but also in hypoxic, weak, and lethargic kids/lambs that are prone to be rejected by the mother due to the lack of neonatal sensorial stimulation.

Uterine rupture and cervical and vulvar tearing are also consequences of dystocia that might disrupt mother-young bonding. In the first instance, uterine rupture is a direct complication of dystocia (e.g., uterine torsion) or is due to human manipulation during parturition (112). In horses (*Equus caballus*), rupture occurs during stage II of foaling (113). Partial or total spontaneous vaginal rupture has been reported in ewes by Mosdøl (114). All ewes presented a dorsolateral tear in the vagina, especially those carrying twins, and approximately one week before lambing. Since most cases are related to uterine torsion (114), ewes can die shortly after the rupture due to circulatory constraints (hemorrhage and shock) (115). Although a single clinical case, this has also been studied in dogs, in whom a Great Dane bitch was presented with uterine rupture and septic peritonitis after manually assisted whelping (116). Due to the severity of the case, all neonate puppies were weaned and subsequently died, although they were hand-reared. In the case of cervical and vulvar tearing, these alterations are associated with the fetus passing through the birth canal (117). Visceral herniation, peritonitis, hemorrhage, and shock might also be present, possibly leading to a weak mother –or even death– (112). These alterations trigger further systemic responses and exacerbate pain, interfering in the establishment of the young-mother bond.

Dystocia also causes metabolic imbalances such as hypocalcemia, hypoglycemia, and hyperlactatemia. Hypocalcemia occurs when blood calcium concentrations are below 10 mg/dL in ewes, does, and cattle during the peripartum period (118). Calcium availability is required at the neuromuscular junction to release acetylcholine (119). However, sustained myometrial contractions cause skeletal muscle fatigue and the depletion of calcium reserves. In the absence of acetylcholine, the transmission of nerve impulses to the muscles is impaired (119). Thus, calcium deficiency causes parturient paresis due to neuromuscular dysfunctions (flaccid paralysis), particularly in ruminants (119, 120). Bendixen et al. (121) reported an association between parturient paresis and dystocia in Swedish Friesian and Swedish Red cattle, particularly in primiparous animals. Contrary to cattle, Bayoumi et al. (118) mention that goats show hyperesthesia and tetany. Due to parturient paresis, the mother is unable to move, staying in recumbency and compromising the interaction with the newborn.

Hypoglycemia is an effect of HPA axis activation and cortisol release (65). This is often observed in small ruminants (122), while it is rare (5%) in bitches but is a factor related to dystocia (123). In rabbits does, hypoglycemia is also related to uterine inertia, another factor that causes maternal exhaustion (124). Due to negative energy balance and fat mobilization, females are susceptible to metabolic homeostasis disturbances exacerbated by decreasing energy supply (122), leading to metabolic acidosis and hyperlactatemia. Tharwat et al. (125) reported alterations in the acid–base balance and blood profile during dystocia in goats. Goats with difficult kidding showed the lactate concentrations significantly higher (4.4 ± 2.5 mmol/L) than those observed in animals with eutocic kidding (1.4 ± 0.5 mmol/L). Additionally, blood pH and HCO_3_ were lower in goats with dystocia than in eutocic animals (7.33 ± 0.18 and 18.9 ± 5.0 mmol/L vs. 7.41 ± 0.05 and 26.2 ± 6.2 mmol/L). In the same species, dystocia significantly elevated lactate dehydrogenase levels (4.24 mmol/L) and decreased HCO_3_ concentrations (28.41 mmol/L), indicating oxidative stress and metabolic acidosis in Damascus goats (126). In dairy cows, Abdela and Ahmed (127) mention that hematological and metabolic changes due to dystocia involucrate stress, exhaustion, or pain, factors that might reduce or delay maternal care.

### 3.4 Aggression towards the newborn

Failure to establish normal maternal bonds might also include refusal to care for the offspring, abandonment, and maternal aggression towards the newborn, including infanticide and cannibalism (128). Aggression towards the offspring is often motivated by increased litter size, reduced food supply (129), or peripartum stress (e.g., dystocia), as mentioned by Zaccarelli-Magalhães et al. (130) in a maternal separation model in rats. The authors highlighted those females separated from their litter for twelve consecutive days increased anxiety and stress (increased serum corticosterone concentrations) and decreased maternal care. These alterations were related to neurochemical dysfunctions in the prefrontal cortex and hippocampus.

**Figure 6:**
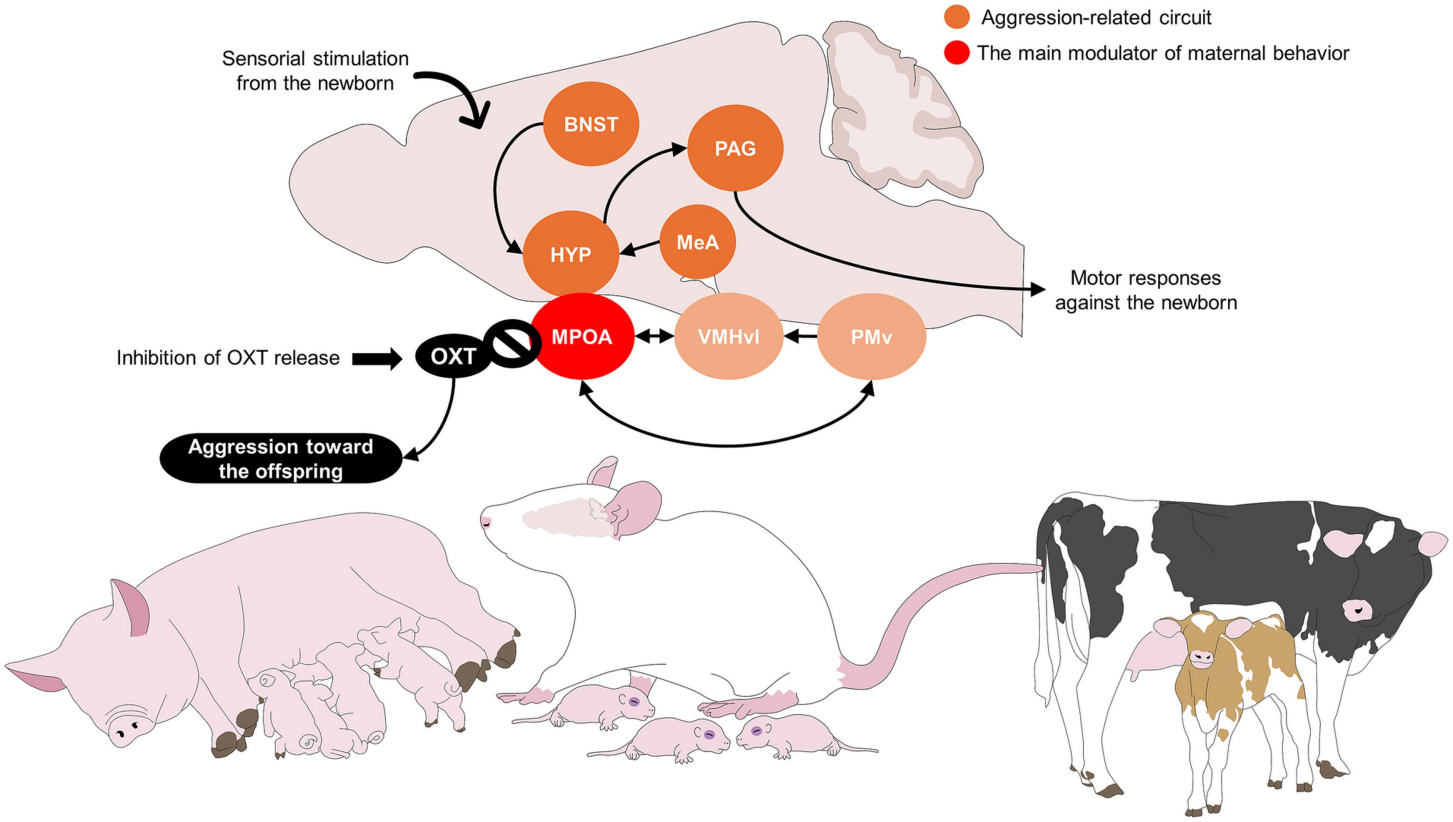
Suggested neurobiology of aggression towards the newborn. OXT is the main mediator of maternal behavior. Thus, the inhibition of OXT signaling in the MPOA is associated with aggression towards the newborn immediately after birth. BNST, bed nucleus of the stria terminalis; HYP, hypothalamus; MeA, medial amygdala; MPOA, medial preoptic area; OXT, oxytocin; PAG, periaqueductal gray; PMv, ventral part of the premammilary nucleus; VMHvl, ventromedial hypothalamus.

Bosch (2) mentions that OXT and the neuropeptide arginine vasopressin modulate peripartum anxiety and, thus, maternal aggression. In mice, a species where 60–90% of females kill unrelated pups, McCarthy (131) reported that subcutaneous and intracerebroventricular administration of OXT to wild mice reduced infanticide from 90 to 18% (Figure 6) (132, 133). Likewise, in Long-Evans rats, Champagne and Meaney (134) reported that stress during gestation reduced OXTr binding at six postpartum days in the BNST, MPOA, lateral septum, and central nucleus of the amygdala, finding an average of 24 fmol/mg in the stressed animals, and up to 40 fmol/mg in non-stressed rats. In cattle, Orihuela et al. (135) emphasize that the presence of predators near the calving site is a factor that might affect the mother-young bonding and elicit aggression towards the calf or reduce maternal responsiveness. Moreover, the frequency of pup licking decreased in stressed animals, from 14 to 10%. These findings suggest that the mother’s endocrine profile can decrease maternal responsivity.

Another example is savaging in gilts and sows, known as the killing or fatally injuring of newborn piglets by their mother. Most studies related to “savaging” are restricted to gilts due to maternal inexperience influence plays on maternal care (71, 128). Harris et al. (71) found that 3.40 and 1.22% of gilts and sows, savaged one or more piglets, respectively. The incidence of savaging in pigs is related to environmental conditions that might be stressful for the animals, such as auditory and illumination (136). Similarly, in Chinese Erhualian and Duroc pigs, the incidence of savaging was around 6.8–14.6% for gilts and 3.2–6.25% for sows (128). Although the etiology of savaging is not well understood, some authors mention that it might be related to hormonal imbalances (e.g., high plasma progesterone and low plasma OXT concentrations) (137, 138) or the environment. According to observations of sows during farrowing, savaging is also related to stress or restlessness (128).

Perinatal stress is another element that affects not only maternal care but also the social behavior of the offspring, as described by Lee et al. (139) in pregnant female rats, in whom stress reduced the level of OXT mRNA. Similarly, the endocrine periparturient profile influences behavioral patterns (1). In dogs, similar results have been found, where dystocic bitches had higher cortisol concentrations postpartum (95). Therefore, complications at parturition can affect the interest of the female in the newborn due to pain, stress, and exhaustion.

### 3.5 Genetic, nutritional, and prenatal factors

Other factors related to poor maternal performance are genetic polymorphisms dictating maternal behavior in several species. For example, in Chinese Hu ewes, Wang et al. (140) reported that polymorphisms in the prolactin receptor gene (PRLR) highly influence licking and kicking behavior. When comparing genotypes AA, AB, and BB, animals with the genotype AA recorded the highest association for licking (715.88 ± 17.20) and suckling (392.75 ± 11.86), and the lowest for kicking (2.75 ± 0.63) and rejecting suckling (5.50 ± 1.19). In contrast, ewes with the BB genotype had the highest kicking and rejecting suckling associations. A better maternal performance was also observed in Blonde d’Aquitaine and Limousine cows (141). Two (NPY1R and ADRA2A) and 56 quantitative trait causative loci were found in these animals, respectively. These loci were related to dams actively stimulating the newborn to suck by licking immediately after calving. Moreover, in Labrador dogs, Ogi et al. (142) reported that single-nucleotide polymorphism (rs8679684) of OXTr (AA+AT) is associated with higher licking (15%) and amount of contact with the pup (2.4%), while correlating with lower levels of sniffing (2.8%). Therefore, these studies show that genetic aspects, in particular polymorphisms of genes involved in endocrine pathways (see paragraphs 3.1 and 3.2), can influence the establishment and success of the mother-young bond.

The mother’s nutritional state before and during parturition is another aspect that might interfere with the bonding process. This has been reported in C57BL/6 J mice fed a high-fat diet (HFD) during pregnancy. At parturition, females displayed more episodes of cannibalism, and the neural activity of the olfactory bulb was reduced, a key structure for the olfactory recognition of the filial pup (143). Similarly, outbred CD-1 mice receiving HFD during the prepartum period presented impaired nest-building behavior and pup retrieval (144). Also, undernutrition in rats decreases maternal attentiveness by delaying pup retrieval (323.38 vs. 57.25 s) and increasing time in active exploratory behaviors (145). This might represent those pregnant females prioritize feeding during the post-partum period over newborn care, which might hinder the establishment of the mother-young bond.

Nutritional issues and predisposition to infections during parturition affect the mother-young bond and the mother’s health. For example, body condition (BC) (over-conditioned sows) at the time of farrowing is related to mastitis-metritis-agalactia syndrome or postpartum dysgalactia syndrome (PPDS). In addition, inadequate husbandry, poor hygiene, and other management-related factors can contribute to the development and severity of PPDS (146, 147). PPDS is characterized by mastitis and endometritis. Sows tend to lie on their painful teats, refusing access to milk. This negatively affects piglets due to an insufficient milk supply (147, 148). The systemic inflammation (due to septicemia and toxemia) state of the sow causes fever, anorexia, and a lack of responsiveness, together with other endocrine alterations (high cortisol concentrations) (149, 150). Additionally, sows with PPDS have behavioral alterations such as reduced nesting behavior and a shift from active to passive behaviors at farrowing (149). PPDS is also related to housing and climate conditions, highlighting the importance of environmental factors on the mother-young bond and the presentation of maternal behaviors, which will be discussed below.

One of the most common causes of disrupted maternal care immediately after parturition is heat stress. Heat stress is defined as when high ambient temperatures are above the upper critical temperature for animals (30–45°C for mammals, although it depends on the species) (151). In Murciano-Granadina dairy goats, Coloma-García et al. (152) have reported that a high temperature-humidity index (85 ± 3) shortened gestation and reduced the number of udder sniffs by the kid. In cattle, heat stress induces behavioral changes such as avoiding direct sunlight and seeking shade (153). Similarly, in sows, temperatures of 25°C resulted in spending less time nest building (25°C = 6%; 15°C = 11%) (154), which shows the effect thermal conditions might have on maternal care.

On the other hand, the environment (e.g., pen, crates, barren enclosures, enriched enclosures) is another aspect that has been shown to affect maternal responsiveness. For example, Lv et al. (155) found that small-tail Han sheep housed in larger pens (6.0 × 3.0 m) increased maternal behaviors such as grooming, suckling, and following. Similarly, providing enrichments to sows (feeder, rope, hose, straw, coconut) shortened the contact time with the piglets during farrowing (1273.5 s). It decreased the time standing up (35,704 s), which increases the chances for successful suckling from the piglets. In full-sib BALB/cJ and Swiss Webster mice, providing nesting materials (disposable polypropylene caps/ hairnets and 3 g of cotton) increased the frequency of licking (1.72 ± 0.20) and nursing in the arched-back position (9.63 ± 0.89). In addition, maternal behavior can be enhanced or perturbed according to the presence of predators, as reported in Long-Evans rats exposed to cat odor on the parturition day (156). Predator odors increased the frequency of licking, grooming, and arched-back nursing. Thus, not only do maternal inherent factors influence the success of the mother-young bond, but environmental elements as well.

## 4 Young-related aspects of impaired maternal bonding

The mother-young bond requires the engagement of both the female and the newborn immediately after calving. An example of young-related aspects associated with a deficient maternal bond is neonatal hypothermia. Mammals have limited thermoregulatory capabilities at birth, particularly altricial newborns (14, 157, 158). Most domestic mammals perform licking to stimulate the newborn’s movement, urination, and defecation, prevent heat loss, and facilitate drying from amniotic fluids (1, 159). This encourages the newborn to stand up and start colostrum intake (160). However, when the mother rejects the offspring or the newborn is not responsive, body heat loss can exceed heat production, significantly dropping the newborn’s body temperature (161, 162). Hypothermia is linked to excessive heat loss or hypoxia-induced, starvation-induced inhibited heat production (161).

During parturition, mammals are exposed to an environment 10–15°C lower than the intrauterine temperature (163–165). Hypothermia is not limited to a decrease in the body temperature of the offspring but also has metabolic implications due to the depletion of glycogen reserves, which might cause adynamic and low neonatal vigor (166–168). When the newborn cannot stand up and consume colostrum, thermogenesis is compromised due to the lack of nutrients and calories that can be obtained directly from colostrum (165, 169). Studies in lambs performed by Nowak et al. (170) reported that close social contact with the mother, by nosing and nuzzling the udder and suckling, promoted maternal recognition within the first 12 h. Likewise, it was observed that suckling increased peripheral (from mean basal concentration of 16.08 ± 0.87 pg./mL to up to 35 pg./mL) and central (cerebrospinal fluid) (from mean basal values of 106.5 pg./mL to 153.9 pg./mL) OXT concentrations.

Moreover, hypothermia might arise due to intrapartum hypoxemia during dystocia (171, 172). Dystocia often leads to acidosis, meconium aspiration, respiratory distress, and failure of passive transfer (173, 174). Thus, prolonged asphyxia in utero or during delivery causes a cascade of metabolic problems that weakens the fetus or newborn, compromising their adaptability to extrauterine life and decreasing their viability and vitality (175–177). Vitality describes the newborn’s vigor during the first hours post-parturition (172, 178). It can be influenced by the physiological immaturity of the newborn, among other factors. Low scores are associated with congenital abnormalities, low birth weight, and higher mortality rates (179, 180). Moreover, vitality in early postnatal life is affected by maternal care (3, 46). In dog puppies, it has been reported that maternal rejection might cause neonatal hypoglycemia due to a lack of nursing (110). Dystocia also increases the activation of stress-mediated pathways, as shown in dystocic dairy calves, in whom salivary cortisol concentrations were higher (approximately 10 ng/mL) than in animals with natural calving (2 ng/mL) (181).

The release of catecholamines is also related to pain and stress responses during lambing. This was studied in gestating ewes by isolating the ewes for two hours (182). When evaluating uterine blood flow and an adrenergic block with labetalol to determine the influence of catecholamines on the fetus, it was found that fetal cortisol concentrations were 8.1 ± 2.1% higher than in the dams. Moreover, an increase in noradrenaline concentrations and a lower uterine blood flow (by 22%) were reported. These alterations caused a shift to anaerobic metabolism and fetal hypertension. The authors highlighted the role of the endocrine control of parturition since the adrenergic block prevented the reduction of fetal blood flow (182).

The vigor and behavior of the offspring are assessed using some neonatal behavioral and reflex parameters, such as searching for the mammary gland, sucking/swallowing reflexes, and righting reflexes (180, 183, 184). Gonzalez-Lozano et al. (185, 186) indicate that piglets born to dystocic sows have a longer latency to first contact with the udder and lower vitality scores than piglets born in a normal farrowing (eutocic). Therefore, low-vitality newborns are not able to establish a normal mother-young bond, which increases the risk of mortality (42). In addition, Dhaoui et al. (187) mention that lamb vigor is related to the season. For example, winter is associated with a slower time to extend their legs (up to 15 min), stand up (approximately 40 min), and reach the udder (50 min). However, maternal care by grooming had a higher intensity during winter than in summer (approximately 30 vs. 20 min, respectively).

When the offspring experiences dystocia at birth, it might also develop meconium aspiration syndrome (MAS) (188, 189). Dystocia compromises fetal circulation, resulting in fetal hypoxia that shifts blood flow to key organs (e.g., heart and brain) (173, 190, 191). Reduced intestinal perfusion increases peristalsis and reduces anal sphincter muscle tone, resulting in the passage of meconium into the amniotic sac (173). Premature passage of meconium creates few or no feto-maternal problems. Still, if fetal hypoxia is severe and persistent, the newborn initiates strong inspiratory movements with an open glottis, allowing aspiration of meconium-contaminated amniotic fluid into the lungs (189). Meconium aspiration causes airway obstruction, which prevents adequate ventilation and also promotes chemical degradation of alveolar surfactant and subsequent neonatal atelectasis and inflammation (173, 192).

Thus, the factors that reduce the newborn’s vitality, activity, and health interfere with the normal development of the mother-young bonding, which has severe consequences for the offspring.

## 5 Alternatives to improve and promote mother-young bonding

Addressing poor maternal behavior is essential to reduce mortality risk in domestic mammals during the first hours after birth (73). Interferences immediately after parturition (e.g., human intervention) are critical for developing the maternal bond (193). In cattle, five minutes of interaction immediately after calving is required so the dam recognizes the calf. In contrast, separation for five hours is related to maternal rejection (50% of the cases) (7, 194). Contrarily, Regueiro et al. (195) found that programmed parturition assistance in ewes improves maternal behavior by decreasing lamb desertion. In this study, Corriedale primiparous ewes not only recorded shorter lambing duration than ewes with natural lambing (19.2 ± 4.2 vs. 42.6 ± 7.8 min) but also had an earlier onset of grooming and did not abandon any lamb. Moreover, lambs with assisted birth had better performance according to higher O_2_ saturation (97.6 ± 1.0%) and shorter times to suck (36.5 ± 6.7 min). This represents an opportunity, contrary to the belief that human intervention affects the mother-young bond. However, it is important to consider the type of productive systems, as some authors reported that maternal behavior in extensive systems with large areas and low input resources might be affected by human presence (184).

In the case of offspring rejection, an alternative is to provide milk replacement or artificial rearing. Belanche et al. (196) reported that milk replacement provided to lambs did not affect the passive immune transfer. However, the authors highlighted the lack of exclusive bonding with one ewe. Similarly, Napolitano et al. (197) mention that lamb mortality increases to 10–15% in animals artificially reared. Additionally, Love et al. (198) address milk replacement in lambs. Although it might be a practice recommended for animals with maternal deprivation, the authors showed significant brain alterations in mother-deprived lambs. Brain growth and maturation were delayed in these animals, observed as a smaller caudate nucleus and anisotropy of white matter. Another technique to compensate for the failure of mother-young bonding is fostering. This can be performed immediately after birth or by smearing the alien offspring with the amniotic fluids of filial young (199).

In the case of research laboratories and breeding facilities, an alternative to improve the mother-young interaction at parturition is providing elements to enhance maternal performance, such as environmental enrichment. Environmental factors have previously been discussed as altering maternal responsiveness during parturition. Mice and rats, as prey species, are highly susceptible to stress inside laboratories. Thus, studies have shown that enriching animals’ environments with larger and complex cages, nesting materials, or objects to play with reduces anxiety-like behaviors and neophobia, and increases the presentation of arched-back nursing, pup licking, and retrieval (200). Additionally, reducing environmental stressors needs to be considered, as it has been shown in mice that early life stress alters maternal behaviors such as nursing, licking, and contact with pups (201). For rabbits, which could be kept as pets, farm or laboratory animals, some studies have underlined the importance of nesting material for kits’ survival, notably its quality and its abundance (202). The housing system is also of utmost importance: in particular, continuous group housing systems for reproducing females have been definitively proven to challenge animal welfare by increased aggression and injuries among does and to kits, leading to higher mortality rates of kits when females are group-housed compared to individually housed females (203). In this species, providing enrichment material can also enhance the mother’s welfare: for instance, platforms provide mothers with the opportunity to escape from their kids and have a rest when they leave the nesting box (204). Finally, a stressed or frightened doe can jeopardize the welfare and survival of her kits by jumping into the nest, disturbing their sleep or even scattering or trampling them: solutions based on controlled nursing (i.e., limiting does access to the nest box a few minutes per day at the lactation time in the morning) appeared to overcome this issue (205).

Current precision livestock farming techniques are also an alternative to monitor parturition and detect abnormal interactions between the newborn and the mother. For example, remote monitoring devices (e.g., proximity loggers and pedometers) can evaluate cow-calf contact and suckling bouts (1). Kour et al. (206) used proximity loggers and tri-axial accelerometers to determine calves’ suckling behavior and cow-calf. These remote monitoring devices automatically collect information such as the frequency and duration of certain behaviors by attaching loggers to the animal’s neck or ear tag (1).

**Figure 7:**
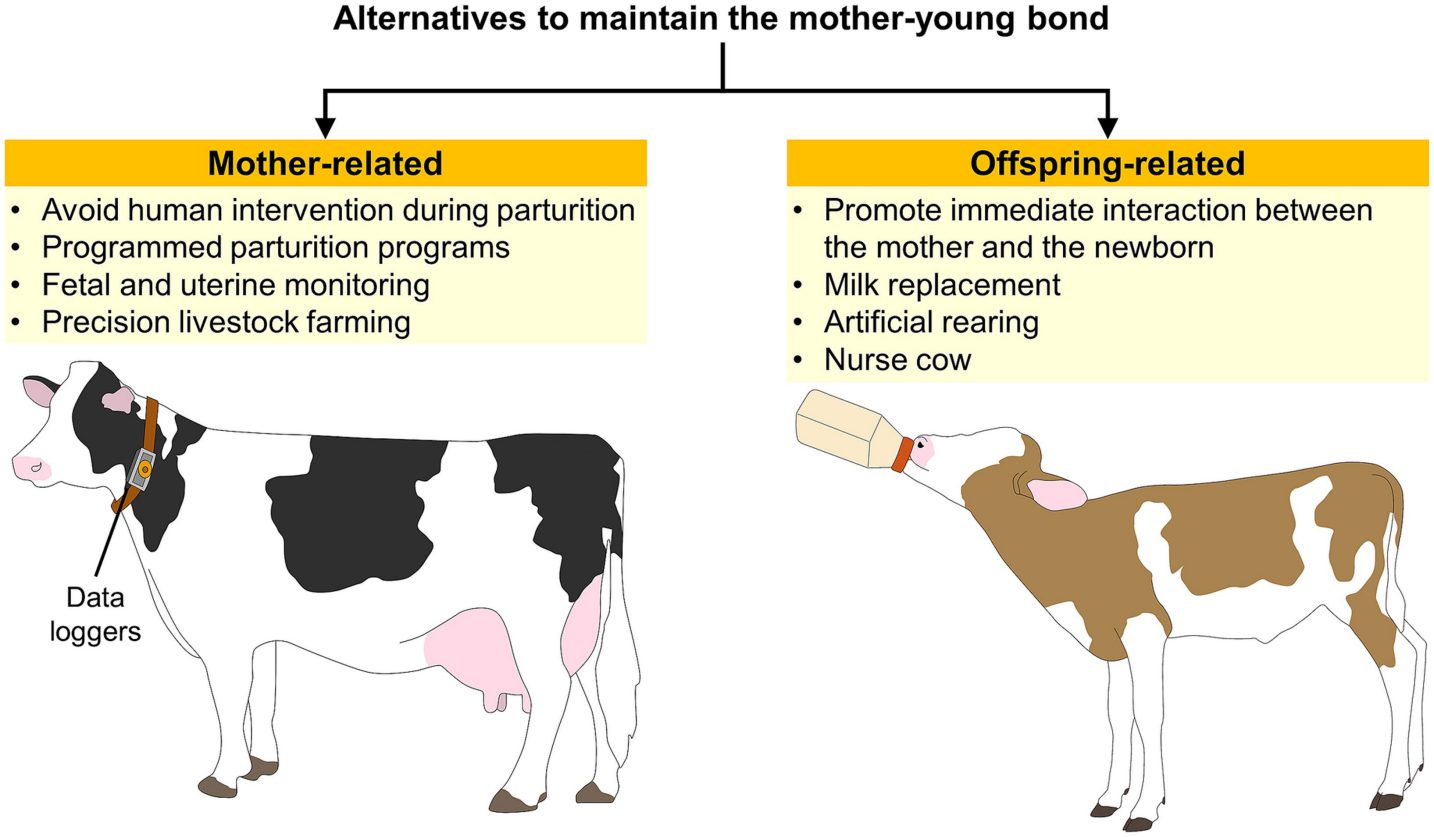
Alternatives to maintain the mother-young bond.

Another alternative could be peripartum monitoring through electronic fetal and uterine monitoring, which can assess uterine dynamics and fetal vitality before and during parturition (207–209). By recording fetal movements, heart rate, and uterine contractions, dystocia could be predicted, and management strategies might be implemented before posing a risk to the newborn and the mother-offspring bond (210). Figure 7 summarizes the current alternatives to improve the maternal bond (1, 196–198, 206).

## 6 Conclusion

The interaction between the mother and the newborn is a selective learning process where the females exclusively care for and protect their filial offspring. Although maternal care is an innate behavior in mammals, several factors can affect and impair the mother-young bond during the first hours after birth. Regarding mother-related factors, alterations in OXT release and OXTr expression reduce key behaviors such as grooming, retrieval of pups, and nursing, among others. When females experience dystocia, this might also result in impaired maternal communication and aggression due to pain. Moreover, parity greatly influences interaction, where primiparous mammals delay maternal care and might result in neonatal mortality.

Regarding the newborn, low vitality scores accompanied by hypothermia, adynamia, and overall low responsiveness to the environment after birth affect the mother-young bond. Precision livestock farming techniques have been implemented in domestic mammals to monitor the peripartum period and prevent the consequences of failed maternal communication. In the prepartum period, uterine and fetal monitoring might help to identify and intervene promptly during dystocia cases. At the same time, proximity loggers and tri-axial accelerometers allow remote behavioral monitoring of the mother and the newborn. Interventions that support natural hormonal and behavioral processes can enhance the success of these programs. For instance, providing environmental enrichments that reduce stress or administering exogenous OXT to promote bonding may be beneficial strategies.
