# Correction: Failure in the mother-young communication in domestic mammals: endocrine and behavioral aspects

**DOI:** 10.3389/fvets.2025.1641847

**Published:** 2025-06-25

**Authors:** Frontiers Production Office

**Affiliations:** Frontiers Media SA, Lausanne, Switzerland

**Keywords:** maternal behavior, newborn rejection, dystocia, oxytocin, maternal care, maternal recognition

Affiliation for Reviewer Natália Cristina Zanta was erroneously given as “Federal University of São Paulo, Brazil”. This should be “Santa Casa de São Paulo, Brazil”.

The original version of this article has been updated.

